# Numerical Investigation of Auxetic Textured Soft Strain Gauge for Monitoring Animal Skin

**DOI:** 10.3390/s20154185

**Published:** 2020-07-28

**Authors:** Han Liu, Matthias Kollosche, Jin Yan, Eric M. Zellner, Sarah A. Bentil, Iris V. Rivero, Colin Wiersema, Simon Laflamme

**Affiliations:** 1 Department of Civil, Construction, and Environmental Engineering, Iowa State University, Ames, IA 50011, USA; yanjin@iastate.edu (J.Y.); ckw@iastate.edu (C.W.); laflamme@iastate.edu (S.L.); 2 Harvard John A. Paulson School of Engineering and Applied Sciences, Harvard University, Cambridge, MA 02138, USA; mkollosche@seas.harvard.edu; 3 Department of Clinical Sciences, College of Veterinary Medicine, Iowa State University, Ames, IA 50011, USA; ezellner@iastate.edu; 4 Department of Mechanical Engineering, Iowa State University, Ames, IA 50011, USA; sbentil@iastate.edu; 5 Department of Industrial and Systems Engineering, Rochester Institute of Technology, Rochester, NY 14623, USA; Iris.Rivero@rit.edu; 6 Department of Electrical and Computer Engineering, Iowa State University, Ames, IA 50011, USA

**Keywords:** flexible sensor, soft sensor, strain, auxetic, texture, biomechanics, soft elastomeric capacitor, polymer

## Abstract

Recent advances in hyperelastic materials and self-sensing sensor designs have enabled the creation of dense compliant sensor networks for the cost-effective monitoring of structures. The authors have proposed a sensing skin based on soft polymer composites by developing soft elastomeric capacitor (SEC) technology that transduces geometric variations into a measurable change in capacitance. A limitation of the technology is in its low gauge factor and lack of sensing directionality. In this paper, we propose a corrugated SEC through surface texture, which provides improvements in its performance by significantly decreasing its transverse Poisson’s ratio, and thus improving its sensing directionality and gauge factor. We investigate patterns inspired by auxetic structures for enhanced unidirectional strain monitoring. Numerical models are constructed and validated to evaluate the performance of textured SECs, and to study their performance at monitoring strain on animal skin. Results show that the auxetic patterns can yield a significant increase in the overall gauge factor and decrease the stress experienced by the animal skin, with the re-entrant hexagonal honeycomb pattern outperforming all of the other patterns.

## 1. Introduction

The study of the mechanical, physiological, and morphological structural responses of skin tissues is of great interest in biomedical fields such as tissue engineering and regenerative medicine [1], and can affect surgical outcomes including the amelioration of scar tissue. The heterogeneous skin is comprised of collagen, elastin fibers, and ground substance in a proteolytic matrix [2], and the study of the mechanical, physiological, and morphological structural responses of skin tissues is of great interest. Recently, different techniques such as optical coherence tomography (OPT) [3] and digital image correlation (DIC) [4] have been used for ex vivo studies of the skin’s deformation. However, these techniques have certain limitations. In particular, OPT is intrusive as it usually necessitates a 2–3 mm probe penetration [5], which is not applicable for thin biological tissues, and DIC technology is difficult to apply in vivo [6]. A solution to quantify deformation is the use of external stretchable and wearable strain sensors. Such technology has been demonstrated for human motion detection [7], health monitoring [8], and soft robotics [9].

Applying sensors to characterize biomechanical behavior and body motion is however not new, and various sensors have been proposed and studied in this field. For instance, sensors constructed with nanoribbons of lead zirconate titanate (PZT) have been applied for the assessment of viscoelastic moduli and spatial mapping [10], passive magnetoelastic sensors implants have been applied to endogenous biological mechanisms [1], thin film-based carbon nanotube strain sensors have been demonstrated for human motion detection [11], and fully polymeric capacitive sensors have been proposed to measure the ligament and tendon elongation at the human knee joint [12]. Of interest to this work is a soft elastomeric capacitor (SEC)-based sensing skin previously proposed by the authors that transduces strain into a measurable change in the capacitive signal [13]. Advantages of the capacitive-based method include (1) low energy required for interrogation, (2) high scalability and large-area monitoring, (3) high mechanical and environmental robustness, (4) high mechanical compliance enabling deployment on irregular surfaces, and (5) customization of shapes and sizes [14,15,16]. The sensor technology has been demonstrated for structural health monitoring applications, notably for the detection and quantification of cracks in concrete structures [17] and fatigue cracks on steel girders [18], in both cases on full-scale components using arrays of sensors.

More related to the application of interest, the capability of the SEC to characterize the biomechanics of canine skin has been demonstrated [6], where the modified Kelvin–Voigt model was used to estimate the stress underneath the sensor. While SEC technology showed promise at quantifying deformation, a limitation is in its lack of sensing directionality that restricts area-wide signal reconstruction capabilities. Furthermore, the SEC technology requires signal decomposition [19] to evaluate the degree of the transverse deformation with respect to the deformation along the loading direction, which is given by the apparent or transverse Poisson’s ratio [20]. Previous work has demonstrated that the Poisson’s ratio of the thin film was tunable by augmenting the sensor with a texture. In particular, a diagrid-like pattern was experimentally shown to enhance directionality and to improve the experimental gauge factor by 30% [21]. The current paper extends the previous study to auxetic patterns, known to yield a negative Poisson’s ratio [22,23]. Frequently used auxetic structures include re-entrant, chiral truss, and semi-rigid rotating patterns [24,25], and they have been applied in the medical field [25], sports [26], aerospace engineering [27], and for the design of piezoelectric strain sensors [28].

This study investigates, for the first time, five auxetic patterns to form textured SECs for tailoring the mechanic inhomogeneities to enhance sensing properties and how they might be described in terms of Poisson’s ratio. The previous study [6] is also extended by applying the textured SEC to characterize the biomechanics of canine skin. To do so, a numerical model of the SEC deployed over the canine skin is constructed and validated using laboratory data collected using a diagrid-like textured SEC adhered onto canine skin. The validated model is used to further our understanding of variations in stress distributions caused by auxetic patterns adhered onto the canine skin layer. Results are compared against those obtained on untextured SECs and the diagrid-like textured SEC.

The paper is organized as follows. Section 2 provides the background on textured SEC and the investigation of auxetic structures. Section 3 describes the methodology used for numerical study and its validation. Section 4 presents and discusses results from the numerical investigation. Section 5 concludes the paper.

## 2. Background

This section provides a background on the SEC technology and the selected auxetic patterns.

### 2.1. Soft Elastomeric Capacitor

**Figure 1:**
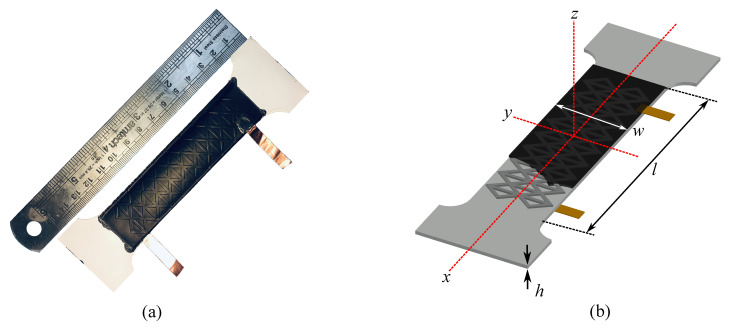
(**a**) Picture of a textured soft elastomeric capacitor (SEC) and (**b**) a schematic of a corresponding SEC of thickness *h* and a section of the electrode layer with electrode area *l* × *w* (black layer).

While the study presented in this paper is numerical, textured SECs (see Figure 1a) were studied experimentally in a previous work [21] to validate the numerical model, and their fabrication is therefore of interest. The SEC consists of a stretchable parallel plate capacitor, where a dielectric layer is sandwiched between two electrode layers. The dielectric is fabricated from a Styrene–Ethylene–Butylene–Styrene (SEBS) matrix mixed with titanium dioxide to adjust the dielectric as well as the durability. The electrode layers are fabricated from SEBS mixed with carbon black particles. This fabrication process, described in more detail in [13], is adapted to create textures by drop-casting the SEBS composite solution in grooved steel molds instead of over smooth glass surfaces.

The sensing principle of the SEC is based on a measurable change in capacitance arising from a change in its geometry provoked by strain. The capacitance *C*, for the SEC at low measurement frequency (<1 kHz), can be written as

(1) $$C=e_{0}e_{r}\frac{A}{h}$$

where $e_{0}=8.854$ pF/m is the vacuum permittivity, $e_{r}$ is the relative permittivity, *h* is the thickness of the dielectric, and A=w·l is the electrode area as annotated in Figure 1b. Differentiating Equation (1), applying Hooke’s Law for plane stress, and taking the in-plane strain expressions $\varepsilon_{x}=\Delta l/l$ and $\varepsilon_{y}=\Delta w/w$, one obtains

(2) $$\frac{\Delta C}{C}=\frac{1}{1-\nu }\left(\varepsilon_{x}+\varepsilon_{y}\right)$$

where ν is the Poisson’s ratio in three principal directions for an isotropic untextured SEC. For a textured SEC, the dielectric layer becomes orthotropic and the Poisson’s ratio in the *x*–*y* plane ($\nu_{xy}$) differs from ν. In this case, under uniaxial stress along the *x* direction, Equation (2) can be written as

(3) $$\frac{\Delta C}{C}=\frac{1-\nu_{xy}}{1-\nu }\varepsilon_{x}$$

where ν is defined as ν = $\nu_{xz}$ = $\nu_{yz}$ and corresponds to the Poisson’s ratio of the untextured SEC. It results that the gauge factor λ of a textured SEC is written as

(4) $$\lambda =\frac{1-\nu_{xy}}{1-\nu }$$

Equation (4) shows that the gauge factor of a free-standing textured SEC is a function of the Poisson’s ratios, and increases with decreasing $\nu_{xy}$. The gauge factor of the sensor will be influenced by its installation, as derived in [29] for an untextured SEC adhered onto an isotropic structural,

(5) $$\lambda =\frac{1-\nu_{m}}{1-\nu }$$

where $\nu_{m}$ is the Poisson’s ratio of the structural material. For the textured SEC, Equation (5) can be written as a function of weighted Poisson’s ratios

(6) $$\lambda =\frac{1-\frac{a\nu_{xy}+b\nu_{m}}{a+b}}{1-\nu }$$

where 0≤a≤1 and 0≤b≤1 are weights such that a+b=1 represents the composite effect, and thus depends on the level of adhesion and material stiffnesses. For a structural material of high stiffness, such as steel or concrete, a≈0 and b≈1, and for the free-standing SEC, a=1 and b=0. Equation (6) can also be written as

(7) $$\lambda =\frac{1-\nu_{xy,c}}{1-\nu }$$

where $\nu_{xy,c}$ denotes the composite effect on the transverse Poisson’s ratio.

**Figure 2:**
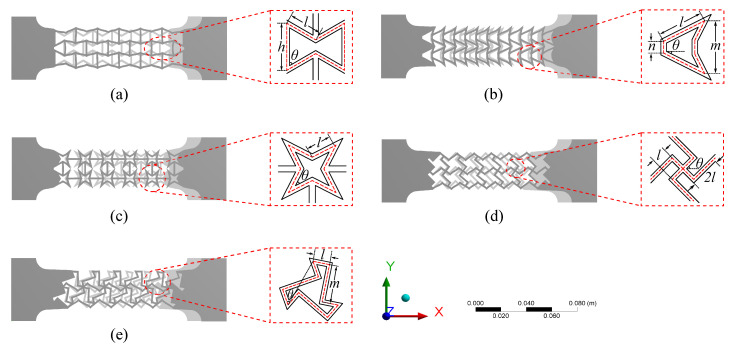
(**a**) Re-entrant hexagonal honeycomb (Pattern A), (**b**) re-entrant triangular-shaped honeycomb (Pattern B), (**c**) re-entrant star system (Pattern C), (**d**) chiral truss (Pattern D), and (**e**) zigzag triangular network (Pattern E).

In this study, λ is increased by decreasing $\nu_{xy}$ using auxetic patterns. For that purpose, five different auxetic patterns are selected and illustrated in Figure 2a–e. Geometries of investigated auxetic patterns are described in what follows, along with the selected geometric parameter values. Note that these values were selected to optimize the negative Poisson’s ratio while respecting geometric constraints from the numerically investigated specimens and enabling a practical fabrication process.

### 2.2. Design of Auxetic Structures

Auxetic structures can exhibit negative Poisson’s ratio [30] due to lateral outwards deformations when stretched. A large number of auxetic structures have been developed, with the re-entrant structure being one of the most common types. The deformation in re-entrant patterns is dominated by the realignment of cell strips (hinging) [22]. Figure 2a–e show the five auxetic structures (Patterns A–E) under study, where the objective is to generate a negative Poisson’s ratio. The rationale for selected these patterns is to be explained in detail next.

- **Re-entrant hexagonal honeycomb (Pattern A)**

The re-entrant hexagonal honeycomb structure [31], also known as bowtie honeycomb, is characterized by the angle θ and cell strip length ratio (β=h/l) (see Figure 2a). The effective Poisson’s ratio $\nu_{xy}$ remains negative and decreases with decreasing θ for 30°≤θ≤80° and increasing length ratio β between 1 and 2 [32]. In this study, Pattern A is formed using θ=60°, l=8.7 mm and β=1.55.

- **Re-entrant triangular-shaped honeycomb (Pattern B)**

The re-entrant triangular-shaped honeycomb is characterized by the angle θ and cell strip length ratio β (β=n/l) (Figure 2b). The negative Poisson’s ratio can be decreased by decreasing re-entrant angle θ for 10°≤θ≤50° and decreasing β for 0.1≤β≤0.3 [33]. In this study, Pattern B is formed with θ=26° to maintain m>n, and using β=0.25 to provide longer shared base connections between two neighboring unit cells.

- **Re-entrant 4-star system (Pattern C)**

The re-entrant 4-star system is characterized by the angle θ and length of the inclined strip *l* (Figure 2c). The numerical simulations performed in [34] have shown that θ=35° yields the lowest Poisson’s ratio and that the Poisson’s ratio remains negative and decreases with increasing *l* for 30 ≤l≤ 70 mm. In this study, Pattern C is formed with θ=35° and l=5.9 mm.

- **Chiral truss (Pattern D)**

The chiral truss is characterized by right-angle crossing strips (θ=45°, Figure 2d), with the longitudinal and transverse strips designated as active and passive strips [35]. The theoretical model characterizing its negative Poisson’s ratio is given in [36]. Here, Pattern D is designed with length l=4.2 mm.

- **Zigzag triangular network (Pattern E)**

The zigzag triangular network is characterized by the intersecting angle θ, length of short strips *l*, and length of long strips *m* (see Figure 2e). The effective Poisson’s ratio has been derived and verified numerically in [30], where it is shown that the Poisson’s ratio is negative and decreases with increasing tanθ for 0 ≤tanθ≤ 1.4, and increasing l/(l+m) for 0.1 ≤l/(l+m)≤ 0.4. Here, Pattern E is designed with tanθ=1.0 (θ=45°) and l/(l+m)=0.3.

## 3. Methodology

This section presents the methodology used for the numerical simulations and experimental tests. First, the geometries, physical properties of materials, and boundary conditions of the numerical model are presented. Second, the experimental procedure for generating validation data for the numerical model is described.

### 3.1. Numerical Models

Two numerical models are generated for the study: The first one is that of a free-standing SEC sensor, and the second of an SEC both partially and fully adhered onto the surface of animal skin. The model of the SEC partially adhered is used to replicate experiments that were conducted on animal skin, and thus to produce a validated model to numerically study the effects of a fully adhered sensor, constituting a more realistic application of the sensor utilized post-surgically.

- **SEC Sensor Model**

Three-dimensional nonlinear finite element models (FEMs) were created in ANSYS 2019 R2 to simulate the SEC sensors. Simulations were set to have specimens with a layer thickness of 0.3 mm and a texture height of 0.35 mm for validation using the experimental data. Material properties, including stiffness and Poisson’s ratio, were both acquired experimentally. The stiffness of an untextured SEC was determined from the slope of the stress–strain measurements on untextured SECs and found to be *E* = 0.41 MPa. The strain rate and strain target were set to 0.1 mm/s and 20%, respectively, during the tensile stretching process.

**Figure 3:**
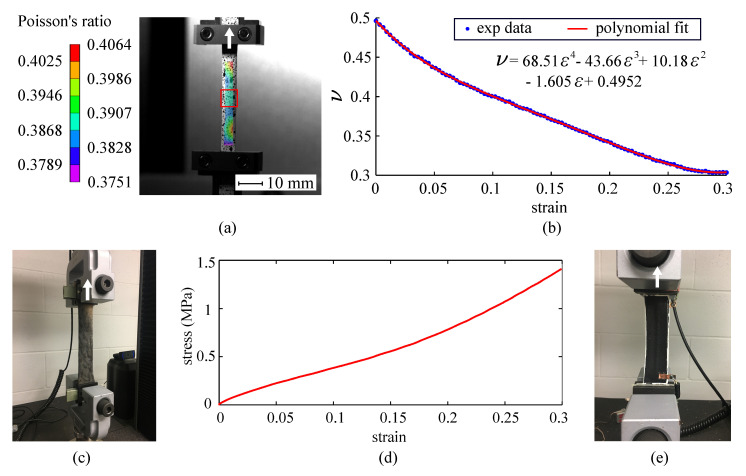
(**a**) Typical digital image correlation (DIC) results showing the Poisson’s ratio distribution under 20% strain, with the region of interest shown as a red rectangle; (**b**) the corresponding transverse Poisson’s ratio curve obtained by averaging values within the region of interest; (**c**) picture of the animal skin subjected to uniaxial strain; (**d**) experimental uniaxial stress versus strain curve of the free-standing animal skin; and (**e**) picture of the free-standing SEC subjected to strain (the arrows illustrates the loading direction).

DIC was used to calculate Poisson’s ratio of the thin film, described in a previous work [21]. Briefly, the Poisson’s ratio ν of an untextured dielectric film was measured with two digital cameras (FASTCAM SA-Z and Photron) by subjecting a specimen to a 30% uniaxial strain applied at 80 μm/s. Note that 30% is the maximum strain level used in this study, consistent with experimental tests conducted on free-standing SECs in [21] and with levels expected for the application [6], and that a characterization of the electromechanical model at higher strain levels is left to future work. A 7 × 7 mm^2^ region around the transverse centerline was defined as the region of interest (red rectangle in Figure 3a) to extract the Poisson’s ratio. The data extraction and analysis was conducted using the DIC software VIC-3D (Correlated Solutions, Inc., Columbia, SC, USA). Figure 3a shows a typical result from the DIC experiment for the specimen subjected to 20% strain, with the colored area denoting the range of Poisson’s ratio values. The Poisson’s ratio in the red rectangle was averaged to create Figure 3b, which plots the experimental averaged Poisson’s ratio as a function of strain. A fourth-degree polynomial was used to fit the experimental data ($R^{2}=$ 0.9952) and integrated into the numerical model to define the Poisson’s ratio behavior.

- **SEC-Animal Skin Model**

**Figure 4:**
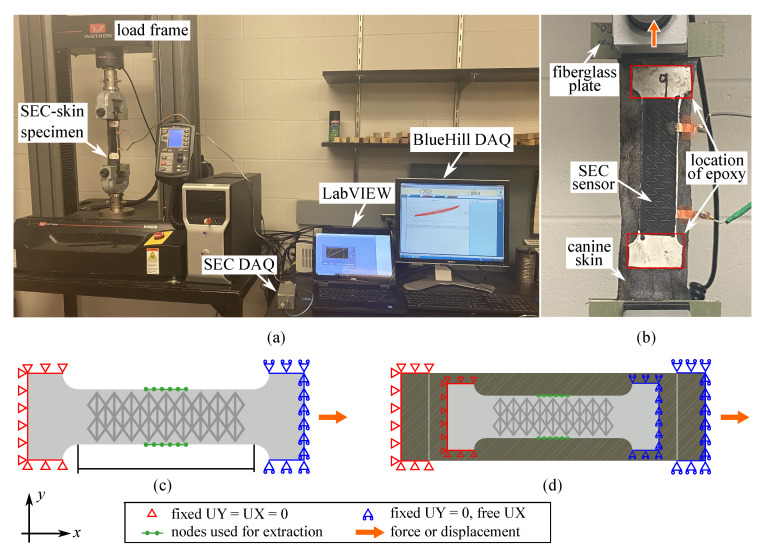
(**a**) Experimental set-up for the SEC-animal skin specimen; (**b**) zoom on the SEC-animal skin specimen showing the loading direction; and (**c**) schematic of the numerical model of the SEC and (**d**) SEC-canine skin showing the boundary conditions, nodes used for synthetic measurements, and loading direction.

The numerical model of the SEC adhered onto animal (canine) skin was generated in ANSYS 2019 R2 by applying the SEC numerical model onto a free-standing animal skin specimen and applying the appropriate boundary conditions to simulate partial or full adhesion. The numerical model of the animal skin was constructed using three-dimensional nonlinear finite elements, with the animal skin specimen measuring 186×64×2.58 mm${}^{3}$. An SEC-animal skin model is shown in Figure 4d.

Similar to other nonlinear FEMs constructed for skin simulations [37,38], the animal skin was assigned to be a nonlinear hyperelastic material with viscoelastic properties. The Poisson’s ratio was taken as 0.43 [39]. Stress–strain experiments were conducted on a canine skin specimen (l×w×h = 186.21×64.62×2.62 mm${}^{3}$) that had been preserved in a euhydrated state (−20 °C) over one year, taken from the animal’s dorsum along the spine. Note that the animal was euthanized for reasons unrelated to this study. The canine skin specimen was soaked in a 0.9% sodium chloride solution for 24 h at room temperature for thawing. The specimen was subjected to uniaxial tensile strain at a 0.01 mm/s rate and 30% strain target. Figure 3c is a picture of the canine specimen under strain. A stiffness value of 0.62 MPa was found for the skin specimen, and the experimentally obtained stress–strain data from uniaxial strain tests (shown in Figure 3d) was imported into the ANSYS database to define the corresponding material properties.

- **Boundary Conditions**

Boundary conditions of the free-standing sensor and partially adhered SEC are shown in Figure 4c,d. The free-standing sensor is assigned as fixed in the *x* and *y* translational degrees-of-freedom (UX and UY) on the left-hand-side, and simply supported in the *y* translational degree-of-freedom (UY) on the right-hand-side. Identical boundary conditions were assigned for the SEC partially adhered onto the animal skin. The tetrahedral and multizone methods were, respectively, applied on the sensor and animal skin model to generate automatic triangular and square meshes, respectively, with an element size of 0.2 mm, which is the maximum size for mesh convergence in this simulation.

Syntectic measurements were taken over 12 nodes set along the two edges of the sensor and skin consistent with the DIC procedure, indicated as green dots in Figure 4c,d, to monitor the different tensile-induced strain contribution in both the longitudinal (*x*) and transverse directions (*y*). The model of the fully adhered SEC onto the animal skin was constructed with identical geometries, boundary conditions, and loading cases, but with the SEC sensor assumed to be perfectly bounded onto the animal skin. Numerical simulations were conducted by applying 30% and 5% axial strain along the right-hand-side support with constant loading rates of 0.3 mm/s and 0.1 mm/s, respectively. The partially adhered SEC-animal skin was also simulated by applying harmonic excitations of 8.05 mm amplitude (5% strain) at 0.1 Hz, and triangular strain inputs, in order to reproduce the experimental inputs and validate the numerical model.

### 3.2. Experimental Tests

The employed numerical model of a free-standing SEC was validated in previous work on several non-auxetic texture patterns [21], and textured sensors were fabricated and tested on animal skin. Here, previous experimental results obtained from a diagrid-like pattern, constructed from intersecting diagonals and vertical reinforcements, are used to validate the numerical model of the SEC partially adhered onto skin. That textured sensor was fabricated as described in Section 2.

Figure 3e is a picture of the experimental set-up with the diagrid-like textured sensor installed in the dynamic testing machine. Both ends of the dog bone specimen were adhered onto fiberglass plates to eliminate sliding and minimize stress concentration. Force–strain experiments of the sensor were performed on two different specimens using an Instron 5969 dual column tabletop equipped with a 2580 series load cell (see Figure 4a). Displacements and axial forces were acquired using a BlueHill DAQ at a 12 Hz sampling frequency. Like the numerical simulation, all tested sensors were pre-strained to 0.5% and strained up to 30% strain using a strain rate of 0.3 mm/s.

The diagrid-like textured SEC was adhered onto canine skin to study the capability of the textured SEC at tracking biomechanical behaviors. The canine skin specimen was taken from the same animal, of the same dimensions, at the same location, and preserved and thawed under the same conditions. The SEC was partially adhered onto the canine skin specimen by applying a bi-component epoxy (JB-Weld) at both ends (under the white area of the sensor, Figure 4b). The SEC-animal skin specimen was also mounted on the dynamic testing machine between fiberglass plates at the clamps, as shown in Figure 4b. The SEC-animal skin specimen was pre-strained by 0.5% before each test, and subjected to a 10-cycle harmonic excitation of 8.05 mm amplitude (5% strain) at 0.1 Hz. Pre-strain was used to ensure that the specimen would remain in tension under lower strain loads and eliminate any slack. Displacements and axial forces were recorded using a BlueHill DAQ at a 12 Hz sampling frequency. Capacitance data was collected at 260 Hz using a customized DAQ operated in a LabVIEW environment.

## 4. Results and Discussion

This section validates the FEMs and presents modifications of the validated FEMs to study the material performance for auxetic patterns through an investigation of the stress distributions and transverse Poisson’s ratios, and to study variations in stress distributions caused by auxetic patterns into the canine skin layer. Results are benchmarked against those from the diagrid-like pattern (Pattern F) that exhibited excellent improvements in sensing properties in previous work [21], and from an untextured SEC (Pattern G).

### 4.1. Numerical Models Validation

**Figure 5:**
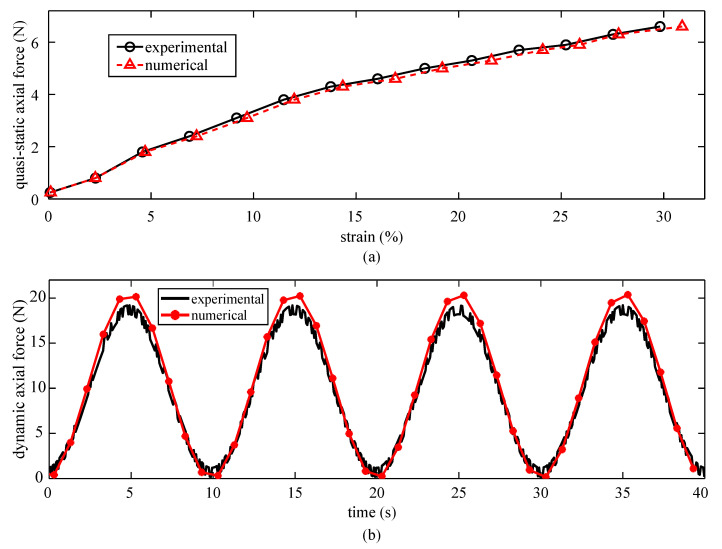
Validation of the numerical models: (**a**) experimental versus numerical quasi-static axial force–strain curves for a free-standing SEC sensor, and (**b**) experimental versus numerical dynamic axial force–strain curves for the partially adhered SEC-canine skin specimen.

Figure 5a compares the axial force versus strain values obtained experimentally and numerically on the diagrid-like patterned SEC. The experimental strain values are averaged, while numerical strains were obtained by extracting and averaging the relative displacements over each node along the *x*-direction under axial forces (indicated as green dots in Figure 4c). The root mean square error (RMSE) between the experimental and numerical values is 1.19% strain, validating the accuracy of the FEM. The independent stress–strain measurements on the dielectric and composite configuration of an untextured SEC were also evaluated. The experimentally measured absolute axial force was equal to the sum of the individual contributions within approximately 5% error. The theoretically calculated values from micromechanics also confirmed experimental results for the moduli.

The validation of the partially adhered SEC-canine skin model was conducted by comparing experimental and numerical axial force measurements from the cyclic test. Results are plotted in Figure 5b for the first four cycles of a triangular loads of 8.05 mm amplitude (5% strain) at 0.1 Hz. Results show a good match between the experimental and numerical data, with an RMSE of 2.06 and a maximum error of 6.32%, validating the accuracy of the numerical model. The discrepancies in results can be attributed to the modeled differences in materials properties between different canine skin specimens and to the unmodeled effects of the adhesive layer.

**Figure 6:**
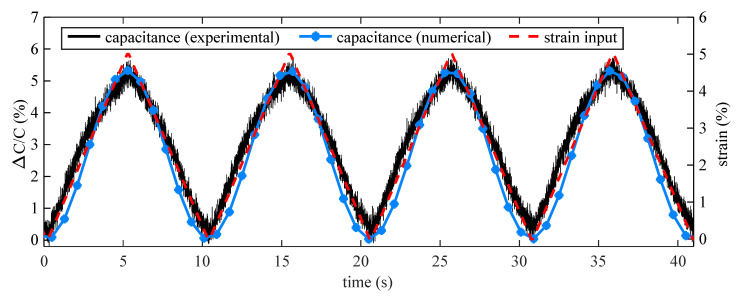
Time series responses of Pattern F adhered partially onto canine skin.

The electromechanical model derived in Section 2.1 is validated on dynamic experiment test data. Figure 6 plots the time series response of the measured relative capacitance for the diagrid-like patterned sensor (Pattern F) partially adhered onto the canine skin under the triangular strain input. Results are compared against those obtained numerically by applying the electromechanical model Equation (7) to the FEM-simulated response. Results show good agreement between the experimental and numerical electrical responses, therefore validating the electromechanical model. Note that the numerical response is lower, attributable to out-of-plane deformations. The tested canine skin sample is itself a three-layer composite that includes epidermis, dermis, and underlying fat [40], and variability in its thickness could cause a reduction in stiffness in the out-of-plane direction and finally lead to out-of-plane deformations.

### 4.2. Free-Standing SEC

- **Stress Distributions**

**Figure 7:**
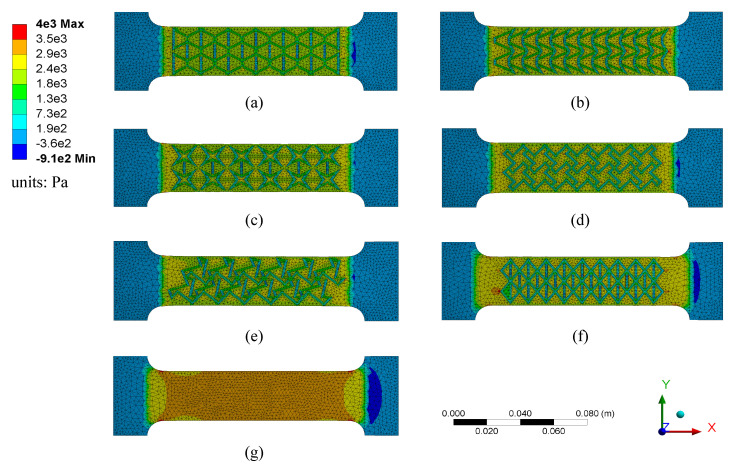
Simulated stress distributions for chosen specimen designs at 10% strain: (**a**) re-entrant hexagonal honeycomb (Pattern A), (**b**) re-entrant triangular-shaped honeycomb (Pattern B), (**c**) re-entrant star system (Pattern C), (**d**) chiral truss (Pattern D), (**e**) zigzag triangular network (Pattern E), (**f**) diagrid-like system (Pattern F), and (**g**) un-textured dielectric (Pattern G).

Figure 7 reports the normal stress distributions obtained numerically from the FEM analysis for the seven different patterns subjected to a 10% uniaxial tensile strain. This strain was generated by pulling the specimens along the *x* axis on the right-end, with the left-end fixed. From the results, it can be observed that (1) both tensile stresses (positive values) and compression stresses (negative values) were generated under the longitudinal strain; (2) due to the inhomogeneity of the textured configurations, compression stresses were mainly concentrated on vertical and vertically inclined strips, while tensile stresses were mainly distributed on the substrate layer; (3) for Patterns A, C, and F, the compression stresses concentrated on vertical strips are higher than those on the inclined strips as they provide higher transverse stiffness; (4) compression stresses exist around the lattice nodes of the zigzag triangular network (Pattern E) that are caused by the clockwise rotation movement under stretch; (5) by comparing the textured specimens to the untextured one (Pattern G), the existence of textures significantly reduced the magnitude of tensile stresses distributed in the substrate layer; (6) by comparing the auxetic textured specimens to the diagrid-like pattern (Pattern F), the overall tensile stress distributions on the substrate layer are lower; and (7) a crescent-shaped stress concentration was formed at the right-hand side of each dog bone due to the asymmetric boundary conditions.

- **Transverse Poisson’s Ratio**

**Figure 8:**
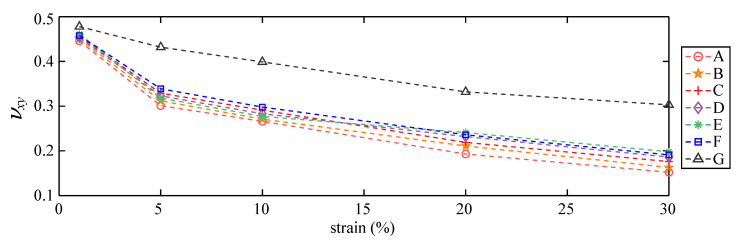
Transverse Poisson’s ratio as a function of strain under all patterns.

The transverse Poisson’s ratio $\nu_{xy}$ was obtained numerically from the extraction and averaging of the relative displacements at each node (green dots, Figure 4c) along the *y*-direction, at 1, 5, 10, 20, and 30% strain. They are plotted in Figure 8. The transverse Poisson’s ratios are found to decrease with increasing strain for all patterns. Among them, results confirm that the auxetic effect yields a lower transverse Poisson’s ratio when comparing Patterns A–E to Pattern F, except for Pattern E yielding a slight increase in the transverse Poisson’s ratio for strain levels higher than 20%. Pattern A maintained the lowest transverse Poisson’s ratio under all strain levels.

The performance of Pattern A in terms of reduction in the transverse Poisson’s ratio is compared against that of Patterns F (best results from previous work on non-auxetic patterns) and G (untextured dielectric) in Table 1. Results show that the auxetic pattern yields substantial gains compared to both Patterns F and G, with a reduction in the transverse Poisson’s ratio up to 16.6% with respect to Pattern F, and 47.8% with respect to Pattern G at 30% strain.

The gauge factors λ for each pattern are computed from Equation (4) using the numerically obtained values for $\nu_{xy}$. Results are listed in Table 2, as well as the average gauge factor $\bar{\lambda }$ and its standard deviation σ under each pattern. Note that λ is expected to be independent of the strain level in order to provide linearity in sensing. Previous work has shown that both ν and $\nu_{xy}$ vary approximately proportionally, and that λ was found to be independent on the applied quasi-static strain level [21]. Thus, σ can be taken as a measure of sensing linearity. Results in Table 2 show that the Pattern A yields the highest gauge factors, as expected from the transverse Poisson’s ratio results, with an increase in $\bar{\lambda }$ of 5.0% and 22.0% compared with Patterns F and G, respectively. In terms of sensing linearity investigated using σ, Pattern F outperforms among the textured patterns, and Pattern C yielded the highest linearity among the auxetic patterns, while Pattern E yielded the lowest. It can also be observed that λ is significantly lower at 1% strain for all of the textured patterns, and slightly decreases under high strain levels. In field applications, a pre-stretch of the sensors may improve sensing linearity.

### 4.3. SEC-Animal Skin

**Figure 9:**
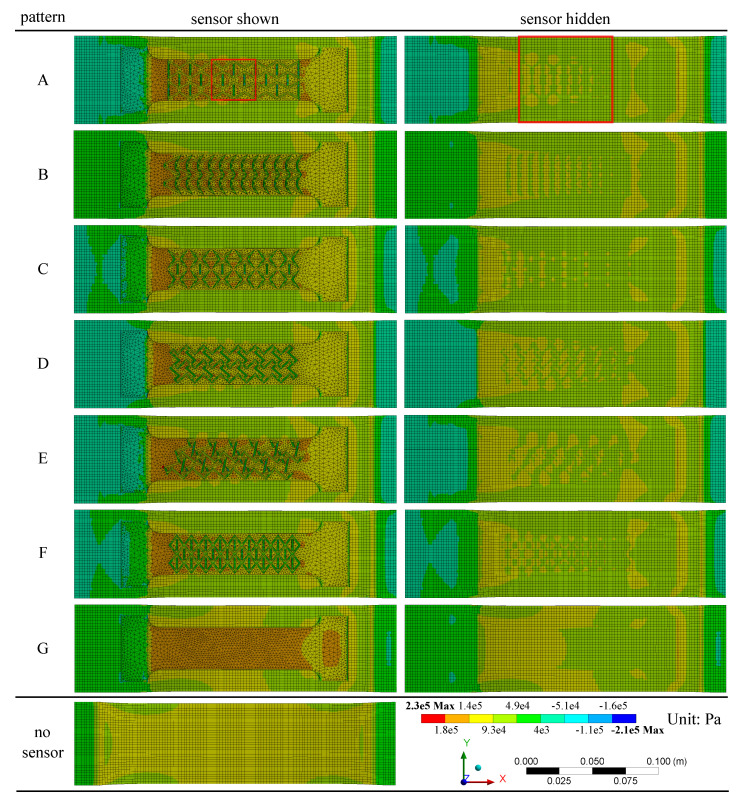
Simulated axial stress distributions for fully attached canine skin model under different patterns at 5% strain, including the pure-skin configuration (no sensor).

A systematic evaluation of the role and impact of the auxetic textured surface on the stress distribution in the animal skin was conducted numerically on the fully adhered SEC-animal skin model, developed from the validated partially adhered SEC-animal skin model. Figure 9 reports the normal stress distributions obtained from the FEM analysis SEC-animal skin subjected to a 5% uniaxial tensile strain for Patterns A–G, along with the canine skin only (no sensor). Results are illustrated with both of the sensors showing and hidden. Table 3 lists quantitative measures from the numerical investigation, including the composite transverse Poisson’s ratio $\nu_{xy,c}$, the corresponding gauge factor λ Equation (7), the minimum stress at the sensor level ($\sigma_{\min,\mathrm{SEC}}$), the maximum stress at the sensor level ($\sigma_{\max,\mathrm{SEC}}$), and the maximum stress at the skin level ($\sigma_{\max,\mathrm{skin}}$). All stress values are normalized to ${\left|\sigma \right|}_{\max}=1$, where the maximum absolute among all stresses was found to be 2.21e5 MPa. The minimum stress at the skin level is not reported as it does not undergo compressive deformations. The composite transverse Poisson’s ratio $\nu_{xy,c}$ was obtained numerically by extracting and averaging of the relative displacements at each node along the *y*-direction (green dots in Figure 4d). Minimum and maximum stress levels were extracted around the center of the specimens, marked as red squares in Figure 9 under Pattern A, corresponding to 32 × 32 mm${}^{2}$ and 64 × 64 mm${}^{2}$ areas over the SEC and animal skin specimens, respectively. These areas were selected to minimize the impact of boundary conditions, consistent with the methodology used for the DIC evaluation.

From Figure 9, the following qualitative observations can be drawn. (1) There is a slight transverse shrinkage generated on the canine skin; (2) the distribution of tensile stress is inhomogeneous on pure canine skin (no sensor), and lower tensile stress formed at the four edges in a crescent shape attributable to out-of-plane deformation caused by a nonuniform thickness of the animal skin layer; (3) the stress distribution on both the sensor and canine skin is asymmetric along the *x*-axis but mostly symmetric along the *y*-axis, attributable to the boundary conditions; (4) compression stresses (negative values) are mainly distributed on the sensor strips along the transverse direction due to the tensile-induced transverse shrinkage; (5) compression stresses are nonuniformly concentrated on the two ends of the canine skin, which could potentially be attributed the curled up out-of-plane deformation; (6) overall, the tensile stress on the sensor was higher than on the skin; (7) higher tensile stress distributions are found on the left-hand-side of all the sensors attributable to the applied uniaxial tension and boundary conditions; (8) comparing with other textured sensors, the stress distributed on the sensor with the chiral truss pattern (Pattern D) is the lowest among all sensors, which can be explained by the lower relative space density of the pattern; (9) by comparing the stress distribution on the canine skin layer, the existence of fully attached sensors partially reduced the magnitude of stress distributed on canine skin; (10) the stress distribution underneath the textured sensors (Patterns A–F) is correlated to the shape of the textured surface, and that lower tensile stress is concentrated underneath the vertical projection (along *z*) of the raised textured strips; and (11) comparing with the textured sensors, the untextured sensor (Pattern G) has more uniform distribution of stresses, yet on average higher over its entire area.

A quantitative comparison between patterns using results reported in Table 3 yields the following observations. (1) The transverse Poisson’s ratios under the composite effect $\nu_{xy,c}$ for all textured patterns are higher relative to those reported for the free-standing SEC configuration (Figure 8), attributable to the higher relative stiffness of the animal skin; (2) the zigzag pattern (Pattern E) saw an increase in its gauge factor relative to other auxetic textures; (3) the increase in the transverse Poisson’s ratios corresponds to a 5% (Pattern G) to 8% (Pattern A) decrease in gauge factors compared to the reported average values for the free-standing SEC (Table 2); (4) compression stresses ($\sigma_{\min,\mathrm{SEC}}$) only occurred on the textured sensors, and tensile stresses were 2% (Pattern D) to 38% (Pattern E) higher on the textured sensors compared with the untextured case (Pattern G); (5) the maximum stress in the animal skin ($\sigma_{\max,\mathrm{skin}}$) is 1% (Pattern D) to 14% (Pattern A) lower under all of the textured sensors compared with the untextured case, and the adhesion of a sensor onto the animal skin resulted in an overall reduction of 16% (Pattern G) to 28% (Pattern A) in its maximum stress; (6) the re-entrant hexagonal honeycomb pattern (Pattern A) yielded the optimal performance by maintaining the highest gauge factor and decreasing the overall stress on the animal skin, which could be a desired attribute in biomedical applications (e.g., measuring strain on wounds); and (7) the maximum stress on the sensor $\sigma_{\max,\mathrm{SEC}}$ for a given pattern is 38% (Pattern G) to 103% (Pattern E) higher on than that on the animal skin ($\sigma_{\max,\mathrm{skin}}$) onto which it is adhered.

## 5. Conclusions

This paper presented a study on texturing a soft elastomeric capacitor (SEC) with auxetic patterns in order to improve the sensitivity and sensing directionality. Five auxetic patterns were investigated: the re-entrant hexagonal honeycomb, re-entrant triangular-shaped honeycomb, re-entrant star system, chiral truss, and zigzag triangular network patterns. This preliminary work was conducted numerically using a finite element model (FEM). That model has been validated in our previous work on structural reinforcement-inspired patterns. The model was modified to study the selected auxetic patterns. The study included an investigation of the stress distributions and transverse Poisson’s ratios, with results benchmarked against those of a diagrid-like pattern that exhibited excellent improvements in sensing properties in previous work, and of an untextured dielectric.

Numerical results on the free-standing SEC showed that the inclusions of vertical and vertically inclined strips lowered the tensile stress distributions in the substrate layer through a higher concentration of compression stresses in the strips. An examination of the transverse Poisson’s ratios as a function of applied strain confirmed that the use of auxetic patterns yielded a decrease in the transverse Poisson’s ratio compared to the diagrid-like pattern, except for the zigzag triangular network pattern under strain larger than 20%. Overall, the re-entrant hexagonal honeycomb pattern exhibited the best performance in terms of reducing the transverse Poisson’s ratio, resulting in an increase in gauge factor of 22% compared to the untextured SEC, and of 5% compared to the diagrid-like pattern. The numerical results indicate that auxetic patterns might serve as a tool to improve and tailor the sensing properties of soft sensors.

An investigation of the effects of the auxetic patterns in a composite configuration in which the sensor was used to monitor strain on animal skin was conducted. Simulations consisted of fully bonding the SEC onto canine skin, and observing the variations in stress distributions across the different auxetic patterns. Results showed that the composite configuration resulted in an overall decrease in gauge factors, as expected, and that all textured sensors provoked a decrease in the maximum normal stress experienced by the animal skin by re-directing some of the stress in the patterns’ ribs. In addition, it was found that the re-entrant hexagonal honeycomb pattern outperformed other patterns by still yielding a higher gauge factor, and also providing the highest decrease (28%) in the maximum stress in the animal skin.

Overall, results from this investigation demonstrated the promise of auxetic patterns at increasing the gauge factor of the SEC, while in some cases substantially decreasing stress in the underlaid substrate. These features can be useful in some biomedical applications, for example, in monitoring strain post-surgery, including applications to the monitoring of human soft tissues. Future work will focus on the experimental validation of results presented in this paper.

## Figures and Tables

**Table 1:** Percentage decrease in Pattern A’s transverse Poisson’s ratio compared to Patterns F and G.

Strain Level					
**Pattern**	**1%**	**5%**	**10%**	**20%**	**30%**
F	2.7	11.2	10.8	15.8	16.6
G	6.8	30.2	33.3	43.9	47.8

**Table 2:** Numerical gauge factors λ as a function of strain levels under each patterns, along with average gauge factor $\bar{\lambda }$ and sample standard deviation (σ) values.

Strain Level							
**Pattern**	**1%**	**5%**	**10%**	**20%**	**30%**	$\bar{\lambda }$	σ
A	1.062	1.231	1.223	1.218	1.208	1.220	0.010
B	1.053	1.211	1.213	1.196	1.198	1.205	0.009
C	1.043	1.182	1.181	1.186	1.180	1.182	0.003
D	1.046	1.192	1.189	1.176	1.168	1.181	0.011
E	1.034	1.202	1.203	1.152	1.142	1.175	0.032
F	1.039	1.163	1.168	1.161	1.156	1.162	0.004
G	1.000	1.000	1.000	1.000	1.000	1.000	0

**Table 3:** Composite transverse Poisson’s ratio $\nu_{xy,c}$, corresponding gauge factor λ (Equation (7)), minimum stress at the sensor level ($\sigma_{\min,\mathrm{SEC}}$), maximum stress at the sensor level ($\sigma_{\max,\mathrm{SEC}}$), and maximum stress at the skin level ($\sigma_{\max,\mathrm{skin}}$) under Patterns A to G. All stress values are normalized to ${\left|\sigma \right|}_{\max}=1$.

Pattern	$\nu_{\mathrm{xy},c}$	λ	$\sigma_{\min,\mathrm{SEC}}$	$\sigma_{\max,\mathrm{SEC}}$	$\sigma_{\max,\mathrm{skin}}$
A	0.357	1.132	−0.661	0.780	0.451
B	0.364	1.119	−0.507	0.914	0.480
C	0.370	1.109	−0.593	0.763	0.457
D	0.372	1.106	−0.552	0.737	0.521
E	0.368	1.112	−0.538	1.000	0.493
F	0.389	1.076	−0.579	0.808	0.501
G	0.441	0.958	0.615	0.725	0.526
no sensor	-	-	-	-	0.624

## Abbreviations

The following abbreviations are used in this manuscript.

SHM	Structural Health Monitoring
SEC	Soft Elastomeric Capacitor
OPT	Optical Coherence Tomography
DIC	Digital Image Correlation
SEBS	Styrene-Ethylene-Butylene-Styrene
ROI	Region of Interest
RMSE	Root Mean Square Error
FME	Finite Element Model
